# Fatigue Behavior of Linear Friction Welded Ti-6Al-4V and Ti-6Al-2Sn-4Zr-2Mo-0.1Si Dissimilar Welds

**DOI:** 10.3390/ma14113136

**Published:** 2021-06-07

**Authors:** Sidharth Rajan, Priti Wanjara, Javad Gholipour, Abu Syed Kabir

**Affiliations:** 1 National Research Council Canada (NRC), Aerospace Research Center, 2107 Chemin de la Polytechnique, Campus de l’Université de Montréal, Montréal, QC H3T 1J4, Canada; priti.wanjara@cnrc-nrc.gc.ca (P.W.); javad.gholipourbaradari@cnrc-nrc.gc.ca (J.G.); 2 Mechanical and Aerospace Department, Carleton University, Ottawa, ON K1S 5B6, Canada; abu.kabir@carleton.ca

**Keywords:** fatigue properties, high cycle, low cycle, linear friction welding (LFW), post-weld heat treatment (PWHT), stress relief annealing (SRA), titanium alloys, Ti–6Al–4V, Ti–6Al–2Sn–4Zr-2Mo–0.1Si, mechanical properties

## Abstract

The use of joints fabricated from dissimilar titanium alloys allows the design of structures with local properties tailored to different service requirements. To develop welded structures for aerospace applications, particularly under critical loading, an understanding of the fatigue behavior is crucial, but remains limited, especially for solid-state technologies such as linear friction welding (LFW). This paper presents the fatigue behavior of dissimilar titanium alloys, Ti–6Al–4V (Ti64) and Ti–6Al–2Sn–4Zr–2Mo–0.1Si (Ti6242), joined by LFW with the aim of characterizing the stress versus number of cycles to failure (S-N) curves in both the low- and high-cycle fatigue regimes. Prior to fatigue testing, metallurgical characterization of the dissimilar alloy welds indicated softening in the heat-affected zone due to the retention of metastable β, and the typical practice of stress relief annealing (SRA) for alleviating the residual stresses was effective also in transforming the metastable β to equilibrated levels of α + β phases and recovering the hardness. Thus, the dissimilar alloy joints were fatigue-tested in the SRA (750 °C for 2 h) condition and their low- and high-cycle fatigue behaviors were compared to those of the Ti64 and Ti6242 base metals (BMs). The low-cycle fatigue (LCF) behavior of the dissimilar Ti6242–Ti64 linear friction welds was characterized by relatively high maximum stress values (~ 900 to 1100 MPa) and, in the high-cycle fatigue (HCF) regime, the fatigue limit of 450 MPa at 10^7^ cycles was just slightly higher than that of the Ti6242 BM (434 MPa) and the Ti64 BM (445 MPa). Fatigue failure of the dissimilar titanium alloy welds in the low-cycle and high-cycle regimes occurred, respectively, on the Ti64 and Ti6242 sides, roughly 3 ± 1 mm away from the weld center, and the transitioning was reasoned based on the microstructural characteristics of the BMs.

## 1. Introduction

Aircraft and aero-engine structural components are subjected to arduous cyclic loading conditions due to repeated flight cycles. Thus, the development of novel design concepts requires validation of the low-cycle fatigue (LCF) and high-cycle fatigue (HCF) behavior, as well as the associated failure mechanisms, so as to ensure structural durability (including the development of inspection and maintenance schedules), reliability and safety. Thus, realizing novel design concepts requires selection of both the material(s) and manufacturing process(es), followed by rigorous testing for certification.

For instance, a relatively recent design concept for aero-engine compressors is the bladed disk or blisk, which was devised for weight-lightening by eliminating the mechanical blade to disk attachments, thereby removing the blade roots and blade-locating slots on the disk. Titanium and its alloys have been the choice materials for the compressor disks and blades of aero-engines due to their structural efficiency for manufacturing such critically loaded components that require a combination of properties, including high resistance to fatigue cracking, high specific strength and excellent corrosion resistance.

Initially, the design for manufacturing titanium alloy blisks conceived the use of material removal technologies to ensure high performance and safety through a monolithic product. However, subtractive manufacturing proved to be not only cost-intensive—because of the low buy-to-fly ratio and related substantial waste of high-cost titanium materials—but also prevented higher fuel efficiency gains from being realized through the application of dissimilar materials that could be tailored chemically, microstructurally and mechanically for the local (and disparate) conditions on the blade and disk. Thus, aero-engine blisk design concepts with a welded assembly emerged, but not without their challenges because the joint between the turbine blade and the disk is the most critically (static and fatigue) loaded region due to the locally high centrifugal forces and thermal stresses [1].

Though titanium alloys generally exhibit good weldability, even the application of high-energy-density fusion welding in a reducing vacuum environment would present performance losses—due to, at best, only grain coarsening in the fusion and heat-affected zones (HAZs), if contamination and solidification defects (such as porosity and underfill) could be prevented [2,3,4,5,6,7,8]—that would compromise the operability/reliability of such critically stressed structures. A key niche technology for manufacturing titanium alloy blisks has been linear friction welding (LFW), a solid-state joining process that can produce sound welds (defect-free) with a joint efficiency—strength of a welded joint with respect to the strength of the base metal (BM)—above 100% and failure in the BM [9,10,11]. This technology is suitable for the assembly of complex part geometries and titanium alloys can be reliably joined without the need for any controlled or shielding atmosphere due to the low process temperatures (considerably below the melting point) and rapid welding times (approximately 2 s).

Much of the research on LFW [12,13,14] has focused on characterizing the metallurgy and static tensile properties of titanium alloy welds, most particularly the alpha (α ) + beta (β) Ti–6Al–4V (Ti64) grade [15,16,17,18]—which is the well-known commercial workhorse of the industry, since it occupies nearly half the market share of titanium products [19]. There have been fewer studies aimed at exploring other titanium alloys [9,20,21,22,23,24,25] or dissimilar combinations of titanium alloys [10,26,27] to emulate welds tailored for the disparate service conditions on the disk (LCF) and blades (HCF and higher service temperatures). Presently, the fatigue test data in the open literature for linear friction welds also remain limited in comparison to static tensile test data; needless to say, the overall data for LFW of dissimilar titanium alloys are even less than those for similar titanium alloy welds.

Currently, in the open literature, the study by Wen et al. [28] has examined the strain-controlled fatigue behavior of LFWed dissimilar joints between two α + β titanium alloys, Ti64 and Ti–6.5Al–3.5Mo–1.5Zr–0.3Si (TC11); their study reported that the fatigued welds exhibited a life comparable to the BMs, with failure occurring on the Ti64 side. Stinville et al. [29] conducted constant amplitude fatigue testing of LFWed Ti64 with dissimilar textures and studied the influence of basal texture orientation on fatigue life and failure. Their work indicated that, in the LCF regime, failure occurred in the HAZ adjacent to the BM having a rolling texture oriented along the loading direction, while in the HCF regime, failure happened more randomly, both in the HAZ and BM having a rolling texture oriented transverse to the loading direction. Kuroki et al. [30] also studied the influence of different LFW pressures on the HCF behavior and reported no significant differences in the fatigue strength of similar Ti64 welds; they also undertook vibratory fatigue testing of a compressor blisk prototype and determined that the welds between the blade and disk did not alter the vibratory properties and met the required HCF strength. However, Yang et al. [31] studied LFW of dissimilar titanium alloys, α + β TC11 and near-β Ti–5Al–2Sn–2Zr–4Mo–4Cr (TC17), and reported that the fatigue properties were influenced by the weld process parameters; specifically, the fatigue limit decreased with increasing pressure and welding time, while, with increasing amplitude and frequency, the fatigue limit initially increased and then decreased. Moreover, a study by Flipo et al. [32] reported that, although the LCF and HCF performance of Ti64 linear friction welds exceeds the minimum design allowable stipulated in AMS4911 [33], when compared to the BM, their performance in the LCF and HCF regimes is lower by 27.9% and 50.4%, respectively, which was further supported by failure of their joints in the weld region. Flipo et al. [14,32] conjectured that the lower fatigue performance of the as-welded (AWed) joints could be improved through parametric optimization or a post-weld heat treatment (PWHT), and, recently, Flipo et al. [14] indicated that the LCF and HCF behavior of Ti64 linear frictions welds in the PWHTed condition surpassed the BM performance. For alloy Ti–6Al–2Sn–4Zr–2Mo–0.1Si (Ti6242), García and Morgeneyer [34] also studied the fatigue performance in the AWed condition and reported lower fatigue strength values (with a couple of early failures), as compared to the BM, which they attributed to remnant pores in the weld originating from contamination on the faying surfaces of the joint prior to LFW. Garcia et al. [11] also recently examined the fatigue strength of dissimilar joints between near-β alloy Ti17 and α + β Ti64 and further highlighted the important role of surface preparation prior to LFW on the static and cyclic performance of the joints; their work also indicated that PWHT was ineffective in reversing weld integrity—and concomitant performance—losses that occurred due to bonding defects from surface contaminants.

Based on these previous findings, the fatigue performance of titanium alloy linear friction welds is sensitive to weld integrity, necessitating not only parametric optimization but also appropriate preparation of the faying surfaces prior to LFW. PWHT seems to be another important parameter for maximizing the fatigue performance of titanium alloy linear friction welds, especially considering the high magnitude of the tensile residual stresses in the AWed joints [35]; disappointingly, most research studies on the fatigue behavior have not examined the effect of stress relief annealing (SRA) or PWHT, which are practiced industrially to mitigate the residual stresses after LFW. With these insights in mind, the present research study was devised to evaluate the performance of a dissimilar joint between two industry-relevant titanium alloys, α + β Ti64 and near-α Ti6242. This combination was deliberated for the higher service temperature capacity of Ti6242 (up to 510 °C) [36] relative to Ti64 (around 350 °C) [37]; Ti6242 also has (relative to Ti64) comparatively higher tensile strength, creep and fatigue resistance, as well as good toughness properties [38]. Previous research by the authors [39] has established the LFW parameters for producing integral (defect-free) joints between Ti6242 and Ti64 and has involved comprehensive characterization of the microstructural and microhardness changes across the dissimilar joint in both the AWed and SRAed states. The present work reports highlights of the microhardness evolution, microstructural characteristics and static tensile properties to provide a basis for the comprehensive results on the fatigue behavior of the dissimilar Ti6242–Ti64 joints after SRA. Cyclic fatigue testing of the SRAed welds and both BMs covered a comprehensive range from the LCF (<10^4^ cycles) to the HCF (10^4^–10^7^) regime, which is of relevance for the disparate conditions on the blade (HCF) and disk (LCF) in service. The fatigue limits of the dissimilar joints and the BMs, as well as the fracture surface features of the joints under LCF and HCF failure, were evaluated.

## 2. Experimental Procedure

**Figure 1:**
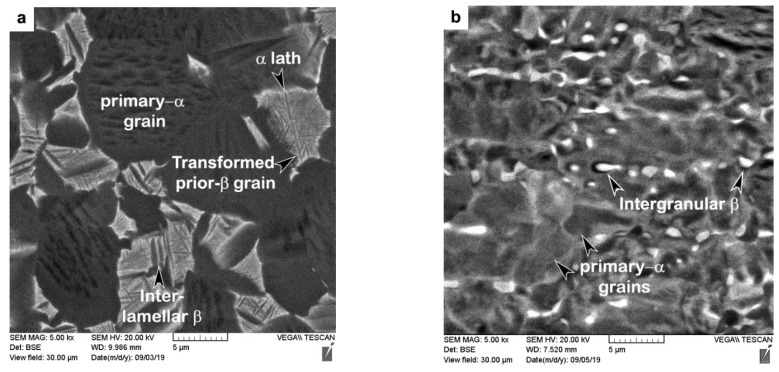
Bimodal microstructures of (**a**) Ti6242 and (**b**) Ti64 BMs.

The as-received AMS 4919 Ti6242 (TIMET, Warrensville Heights, OH, USA) and AMS 4911 Ti64 (TIMET, Warrensville Heights, OH, USA) plates had chemical compositions as listed in Table 1 and bimodal microstructures as illustrated in Figure 1. The Ti6242 alloy consisted of nearly equal fractions of globular primary-α grains and transformed prior-β grains with an average size of roughly 17 µm and 18 µm, respectively. The Widmanstätten secondary α microstructure within the transformed prior-β grains comprised of fine, randomly oriented α and β laths/lamellae, as indicated in Figure 1a. By contrast, the Ti64 microstructure (Figure 1b) consisted primarily of globular primary-α grains with an average size of approximately 12 µm and roughly 6% intergranular β dispersed along the α grain boundaries. Both the Ti6242 and Ti64 alloys also had a rolled texture that is characteristic of mill-plate titanium alloy products processed thermomechanically.

**Figure 2:**
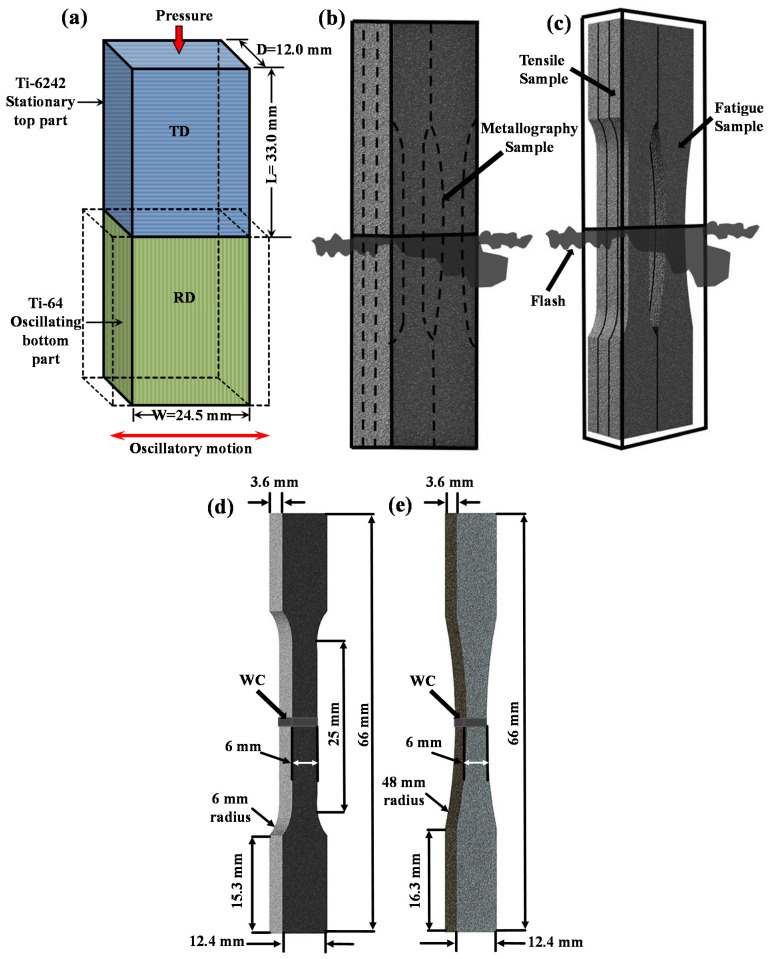
Schematics showing the (**a**) length (L), width (W) and depth (D) dimensions of the Ti64 BM and Ti6242 BM workpieces with the rolling direction (RD) and transverse direction (TD) orientations indicated; (**b**,**c**) Electro-discharge machining (EDM) plan for extracting the tensile, metallography and fatigue specimens from the welded coupons; (**d**) geometry of the tensile specimens; and (**e**) geometry of the fatigue specimens.

Workpieces for the LFW experiments were machined from the 25 mm thick Ti6242 and Ti64 plates to dimensions of 12.0 mm in depth (D) × 24.5 mm in width (W) × 33.0 mm in length (L) with a tolerance of 0.02 mm (Figure 2a). After machining, the contact surfaces of the Ti6242 and Ti64 workpieces were ground manually using 320-grit silicon carbide paper followed by rinsing with ethanol to remove surface contaminants and oxides just prior to clamping within the LFW fixtures. LFW of the dissimilar Ti6242 to Ti64 joints was conducted under standard ambient temperature (25 °C) and pressure (101.325 kPa) conditions, without any shielding gas protection, using a MTS LFW process development system (PDS) (MTS Materials Test Systems, Eden Prairie, MN, USA) at the National Research Council Canada and a set of optimized welding parameters [40] that consisted of an oscillation frequency of 50 Hz, an oscillation amplitude of 2 mm, friction and forging pressures of 90 MPa, a shortening of 2 mm and a total welding time of ~1.3 s. Subsequently, SRA of the dissimilar Ti6242–Ti64 linear friction welds was performed at a temperature of 750 °C and a pressure of 1.6 × 10^−2^ Pa for 2 h in a ceramic-tube vacuum-furnace.

Using electro-discharge machining (EDM) (Fanuc, Oakville, ON, Canada), specimens were extracted, as shown in Figure 2b, for metallographic, tensile and fatigue testing of the dissimilar Ti6242–Ti64 linear friction welds. Metallographic preparation of the dissimilar Ti6242–Ti64 welded specimens involved hot mounting in a conductive resin followed by automated grinding and polishing to a surface finish of 0.02 µm, as described in [39]. Etching for 6–12 s with Kroll’s reagent (100 mL of distilled water, 6 mL of nitric acid (HNO_3_) and 2 mL of hydrofluoric acid (HF)) was used to reveal the characteristics of the different microstructural features (phase constituents, morphology, grain structure, etc.) for microscopic examination using optical microscopy (OM) (Olympus, Richmond Hill, ON, Canada) and scanning electron microscopy (SEM) (Tescan, Warrendale, PA, USA) at 20 keV.

The hardness distribution in the dissimilar Ti6242–Ti64 welds was examined on the transverse cross-sections (i.e., perpendicular to the oscillation direction) of AWed and SRAed specimens taken from the weld center (Figure 2b). Microhardness testing was conducted in accordance with ASTM E92-17 [45] on polished surfaces using a Vickers indenter. Using a Struers DuraScan 80 hardness tester (Struers, Ballerup, Denmark) equipped with an automated cross-travel stage, Vickers microhardness measurements were carried out at a load of 500 g, a dwell period of 15 s and at an indent spacing of 0.2 mm, with this latter selected to ensure that the minimum center-to-center distance between the indents was at least three times the diagonal measurement of the indent. The selected sampling area, 2.5 mm × 7.5 mm (D × L), for hardness mapping sufficiently spanned the entire weldment, i.e., from the Ti6242 side and across the joint interface to the Ti64 side.

Room-temperature tensile testing was carried out on the basis of ASTM E8M-16a [46] using standard sub-size specimens that were machined (Figure 2b) from the AWed and SRAed dissimilar Ti6242–Ti64 joints to a dog-bone geometry, as shown in Figure 2c. The tensile specimens were tested in an MTS servo-hydraulic testing machine (250 kN range) using hydraulic wedge grips and a calibrated laser extensometer at a constant crosshead displacement speed of 0.125 mm/min (with a corresponding strain rate of 5 × 10^−3^ min^−1^) until fracture. The reported tensile properties—yield strength (YS), ultimate tensile strength (UTS) and percent elongation (EL)—are the average of at least three test specimens.

For cyclic testing as per recommended guidelines in ASTM E466-15 [47], fatigue specimens with a dog-bone geometry, as shown in Figure 2d, were extracted from the dissimilar Ti6242–Ti64 joints by EDM followed by machining to a 1 µm finish. The fatigue specimens were tested on an MTS servo-hydraulic testing machine (250 kN range) using hydraulic wedge grips under load control mode with a loading frequency of 6 Hz and a sinusoidal loading waveform. The constant amplitude axial fatigue tests were performed in tension–tension mode at a stress ratio, R = 0.1 (where R is defined as the minimum peak stress divided by the maximum peak stress). To generate a representative fatigue curve that covered both the LCF and HCF regimes, different stress levels from 500 to 1100 MPa were examined. It is noteworthy that initial fatigue tests carried out on the AWed specimens resulted in early/premature failures at a low number of cycles with low-stress amplitudes on the dissimilar joints, which may possibly be due to the internal residual stresses developed during LFW. Thus, further testing of the dissimilar Ti6242–Ti64 welds and the resulting LCF and HCF data reported in the present study involved only the fatigue specimens in the SRAed condition.

To examine the failure mechanisms in the dissimilar Ti6242–Ti64 linear friction welds under tensile and cyclic fatigue loading conditions, fractographic features on the fracture surfaces of the failed specimens were observed using a field emission gun (FEG) SEM (Tescan Vega-II XMU) at 20 keV. The morphology of the fractured surface was examined at low and high magnifications in various locations to identify the crack origin and propagation mechanism(s).

## 3. Results and Discussion

### 3.1. Hardness

**Figure 3:**
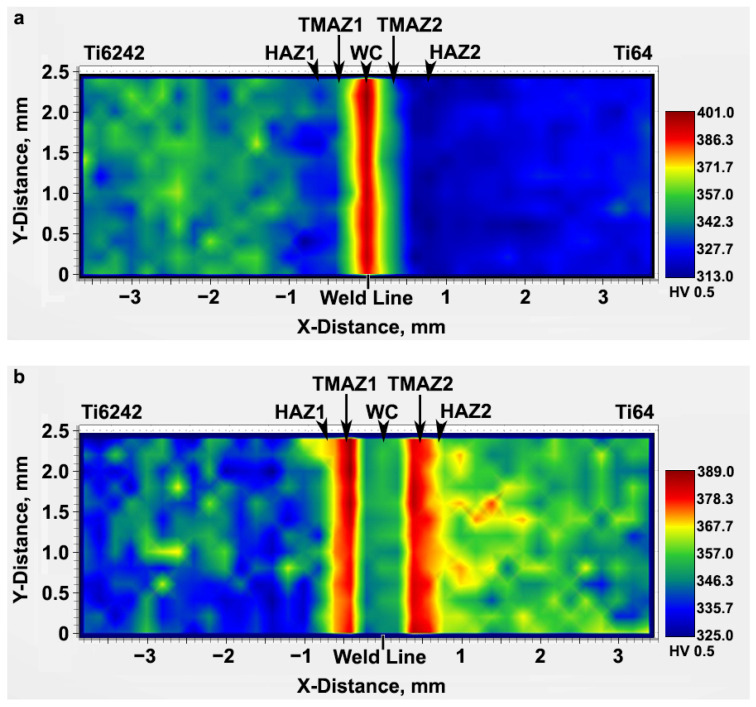
Microhardness maps across the dissimilar Ti6242–Ti64 linear friction welds showing the weld line, weld center (WC), heat-affected zones (HAZs), thermomechanically affected zone (TMAZs), as well as the Ti6242 and Ti64 BMs: (**a**) as-welded (AWed) and (**b**) stress relief annealed (SRAed).

The two-dimensional color-coded maps of Vickers microhardness given in Figure 3 reveal the hardness evolution across the dissimilar Ti6242–Ti64 linear friction welds. In the AWed condition, both the Ti6242 and Ti64 BMs exhibited hardness fluctuations that can be attributed to their complex bimodal microstructures of hard alpha (α) and soft beta (β) phases (Figure 1). The average hardness of the Ti64 BM in the as-received condition was 326 ± 4 HV_0.5_, slightly lower than that of the Ti6242 BM (345 ± 8 HV_0.5_). In the AWed condition, roughly 0.4 mm away from the weld line on the Ti6242 side, softening of the Ti6242 BM by around 4% started and the average hardness was 333 ± 6 HV_0.5_ in this first HAZ (labelled HAZ1) that had a width of 0.6 mm. Similarly, softening of the Ti64 BM by around 3% was observed to start roughly 0.4 mm away from the weld line on the Ti64 side; the average hardness was 315 ± 2 HV_0.5_ in this second HAZ (HAZ2), which was also around 0.6 mm in size. By contrast, hardness peaks were recorded at the weld line separating the two alloys and the maximum hardness in the weld center (WC) was 398 ± 3 HV_0.5_ in the AWed condition. On either side of the WC, the hardness values were lower but higher than in HAZ1 and HAZ2. On the Ti6242 side, the thermomechanically affected zone (TMAZ) lying between the WC and HAZ1, namely TMAZ1, had an average hardness of 370 ± 5 HV_0.5_, while on the Ti64 side, the average hardness of TMAZ2 (between the WC and HAZ2) was 360 ± 3 HV_0.5_.

The SRA treatment had a stabilizing effect on the hardness, as well as the microstructure (as will be discussed subsequently). For instance, as revealed in Figure 3b, SRA at 750 °C for 2 h recovered the softening that occurred in HAZ1 and HAZ2 of the dissimilar Ti6242–Ti64 linear friction welds in the AWed condition. After SRA, Table 2 shows that the hardness of HAZ1 and HAZ2 was, respectively, 10.5% and 13.3% higher than in the AWed condition. By contrast, the hardness in the WC decreased by 7.3% relative to the AWed condition. After SRA, hardness changes in the TMAZs and Ti6242 BM were found to be negligible, but a hardness increase of 8% was measured for the Ti64 BM.

**Figure 4:**
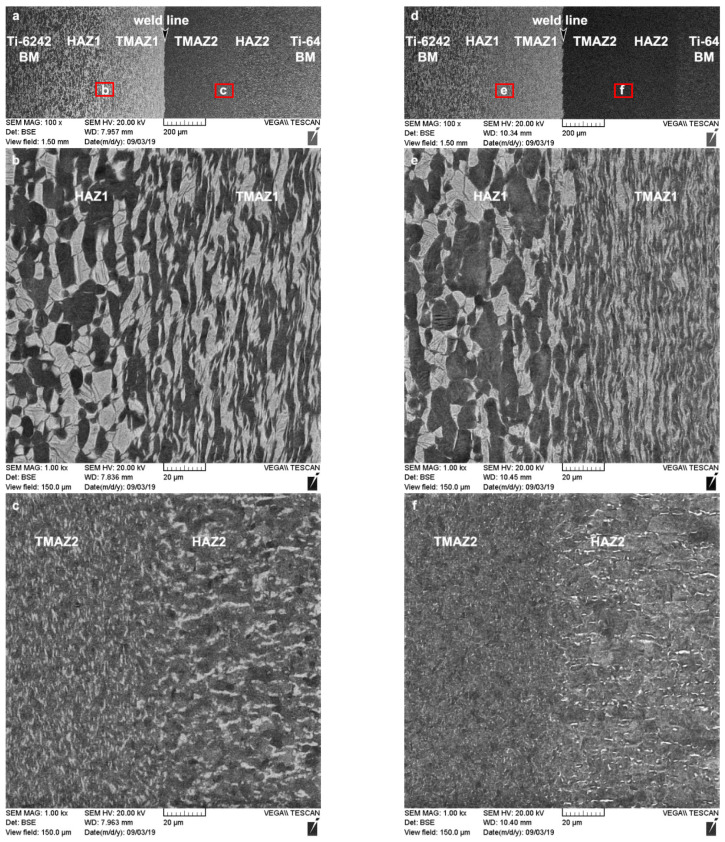
Microstructural characteristics in the different regions of the dissimilar Ti6242–Ti64 linear friction welds in the (**a**–**c**) AWed condition and (**d**–**f**) SRAed condition.

**Figure 5:**
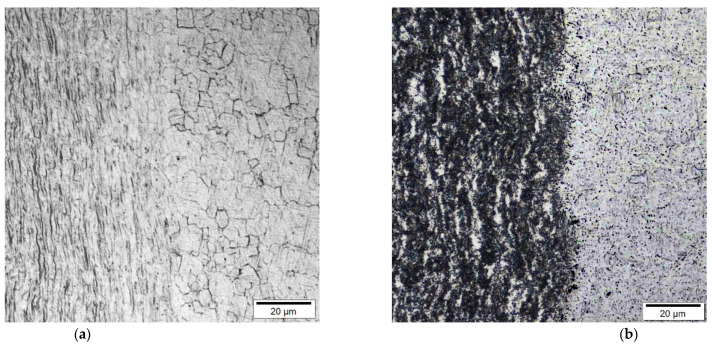
Microstructures in the WC at the weld line in the dissimilar Ti6242–Ti64 linear friction welds: (**a**) AWed condition and (**b**) SRAed condition.

The hardness in the different microstructural regions of the dissimilar Ti6242–Ti64 weld after LFW and SRA is related to the characteristics of the α and β phases, such as the morphology, size, volume fraction, etc. In the AWed condition, the reduction in hardness in the HAZs is attributed to the retention of soft metastable β—the brighter phase in Figure 4a–c—during the rapid cooling conditions after LFW. Diffusion of the β-stabilizing elements (e.g., vanadium and molybdenum) during SRA at 750 °C for 2 h homogenizes and equilibrates the compositional gradients in the bimodal microstructures of the HAZ1 and HAZ2; this favors the transformation of the metastable β phase to equilibrium levels of α and β phases, as shown in Figure 4d–f. Retention of the metastable β phase in the AWed microstructure of the TMAZs also occurred. However, the net effect of metastable β retention on the hardness of TMAZ1 and TMAZ2 is complicated by concomitant hardening in the TMAZs due to the plastic deformation of the bimodal α-β microstructure during LFW, as evidenced by the fragmented and elongated primary-α grains—the darker phase in Figure 4a–c. Thus, even though SRA results in metastable β transformation (which increases hardness), recovery mechanisms (e.g., reduction in dislocation density) reduce the work hardening and hardness. This gives the net effect of statistically insignificant changes in the hardness of the TMAZs after SRA, as tabulated in Table 2. In the case of the BMs, at the temperature selected for SRA (750 °C), changes in the microstructure or the hardness were not expected. However, the as-received Ti64 BM exhibited a hardness increase of 8% after SRA, which was explained (in detail) previously by the present authors in [39], also on the basis of metastable β transformation. Finally, in the AWed condition, the highest hardness values in the dissimilar Ti6242–Ti64 welds were observed at the weld line in the WC due to the formation of α′ martensite within the refined transformed prior-β grain structure (Figure 5a), which is a result of dynamic recrystallization during the high-temperature (>980 °C) deformation combined with rapid cooling after LFW. SRA had the effect of tempering the AWed microstructure (Figure 5b), which led to a reduction in hardness by 7.3% relative to the AWed condition.

Previous studies on LFW of titanium alloy joints (similar and dissimilar) have also applied different microscopic techniques (e.g., optical, SEM) to link the microstructural transformation to the hardness evolution [10,11,15,22,26,27,40,48,49,50]. A particularly favored method for linear friction welds is the use of electron backscatter diffraction (EBSD) to map the orientation of the α-phase grains [13,18,23,26,27,28,51,52]; this method allows visual differentiation of the WC (with its recrystallized fine grain structure of α′ martensite) and the TMAZs (with their plastically deformed and elongated α-grain structure) from the bimodal BM microstructure. As these EBSD maps have focused on the α-phase characteristics, separating the HAZ from the BM has not been obvious microscopically due to the similarity in the α microstructural features. In addition, varying results from hardness measurements have further complicated the situation and prevented a clear resolution of the HAZ, with some researchers detecting localized softening in the BM region adjacent to the TMAZ [15,28,39,40], while others have not [14,29,53]. This disparity originates predominately from the heterogeneous bimodal microstructure of the wrought/forged BM, which includes the different phase constituents and their varying morphologies (lamellar, globular, basket-weave, martensitic, Widmanstätten, etc.), the microstructural texture, as well as the disparate hardness properties of the α and β phases. This, in turn, produces BM hardness differences, which can mask the occurrence of HAZ softening, especially when using linear profiling across the narrow affected regions of the joint (HAZ, TMAZ and WC). The present work provides much clarity on the phenomenon of HAZ softening in titanium alloy linear friction welds. Specifically, it was shown that two-dimensional hardness mapping has efficacy for clearly distinguishing the lower hardness HAZ from the BM, and backscatter electron imaging (BSE) with an SEM permits visualization of the retained metastable β in the microstructure, which is the source of the localized softening. This work also provides insight for fully recovering the hardness through an SRA heat treatment that decomposes the metastable β to an equilibrated α-β microstructure. As this is an existing industrial practice to alleviate high residual stresses in linear friction welds, the SRA heat treatment after LFW serves the additional purpose of also stabilizing the HAZ microstructure.

### 3.2. Tensile Properties

**Figure 6:**
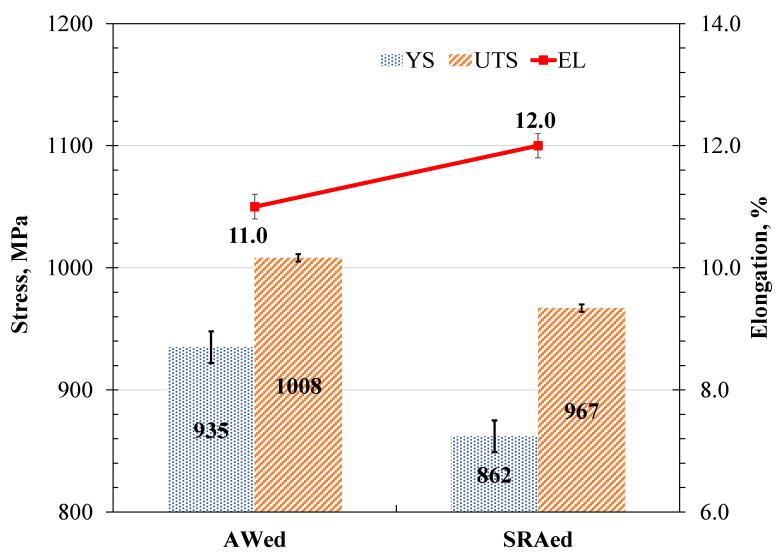
Average tensile properties of the AWed and SRAed dissimilar Ti6242–Ti64 welds.

The results obtained from the tensile tests are represented in Figure 6 in the bar chart showing the stress (MPa) and elongation (%) plotted on the primary and secondary Y-axes, respectively, for the AWed and SRAed conditions of the dissimilar Ti6242–Ti64 linear friction welds. Overall, the average tensile mechanical properties of the AWed and SRAed specimens surpassed the minimum requirements for the YS (~830 MPa), UTS (~900 MPa) and EL (10%), as given in the AMS 4911 specification for Ti64 and AMS 4919 specification for Ti6242. Final failure under tensile loading occurred in the Ti64 BM, around 5 ± 1 mm away from the weld line, indicating the superior mechanical integrity and efficacy of the welded joint region between the Ti6242 and Ti64 BMs.

**Figure 7:**
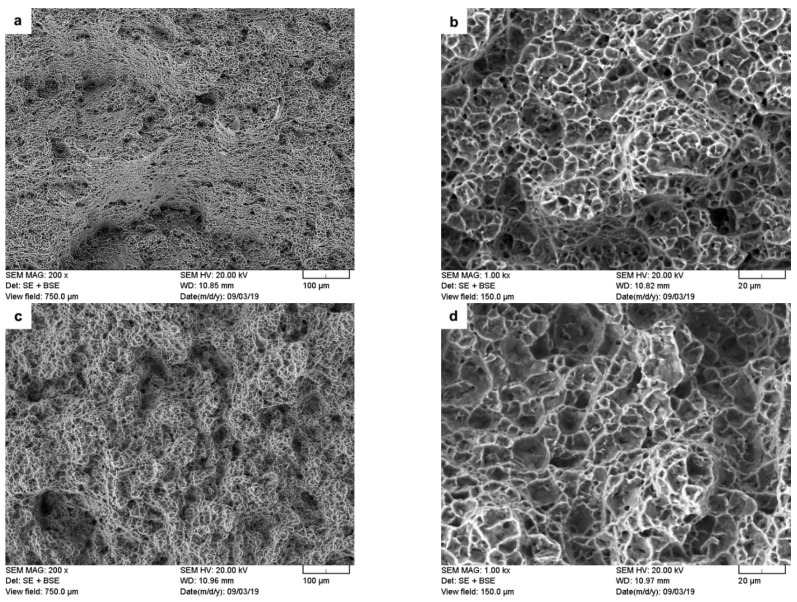
Secondary electron (SE) images of the tensile fracture surface for the (**a**,**b**) AWed and (**c**,**d**) SRAed conditions.

The present findings are in agreement with the research of Corzo et al. [10] on the uniaxial mechanical properties of AWed dissimilar titanium alloy linear friction welds (Ti6246–Ti64 and Ti6246–Ti6242); they reported that the welds outperformed the properties of the BMs. Previously, dissimilar joints between Ti6242 and Ti64 alloys were also studied by Kulkarni and Ramulu [54] using friction stir welding (FSW). The friction-stir-welded joints between Ti6242 and Ti64 were produced using different process parameters, and the YS (790–840 MPa), UTS (870–900 MPa) and EL (1–14%) values [54] were, in most cases, below the AMS specifications of the BMs. Moreover, tensile failure of the friction stir welds occurred predominantly in the weld by either ductile shear or brittle failure, depending on the processing conditions [54]. By contrast, in the present study, the dissimilar joints between Ti6242 and Ti64 alloys manufactured by LFW exhibited tensile properties that surpassed the AMS specifications of both BMs. Furthermore, tensile failure that initiated in the Ti64 BM progressed through a ductile fracture mechanism, as evidenced by the dimples (microvoids) on the fracture surface (Figure 7a,b). These characteristics of the dissimilar Ti6242–Ti64 welds provide reliable assurances of the high weld quality and joint performance, as well as validating the good joining capability of LFW for titanium alloys (including dissimilar combinations).

Table 3 shows the changes in the tensile mechanical properties after SRA of the dissimilar Ti6242–Ti64 linear friction welds. Overall, the strength properties decreased, as evidenced by the reduction in the YS and UTS by 7.8% and 4.1%, respectively. In contrast, the ductility improved with a 9.1% increase in the EL. Moreover, the failure location and fracture mechanism (Figure 7c,d) remained unchanged after SRA, despite hardening of the Ti64 BM (by 8%), as indicated in Table 2, to a value that was statistically equivalent to the Ti6242 BM hardness. This, thus, suggests an influence of the different bimodal microstructures of the Ti6242 and Ti64 BMs on fracture behavior. As mentioned previously, the bimodal microstructure of the Ti6242 BM consisted of globular primary α grains and transformed prior β grains within which the structure consisted of Widmanstätten secondary α-β lamellae (Figure 1a). On the other hand, the bimodal microstructure of the Ti64 BM comprised predominately of globular primary α grains with a small fraction (6%) of fine intergranular β. This latter microstructure, with its high fraction of interconnected primary α (94%), has been associated with a higher predisposition for failure due to the longer effective slip length of similarly oriented α particles [55,56,57]. SRA—which induces a small reduction in the intergranular β fraction (by ~1%) [39]—exacerbates the situation further. This reasonably explains the higher susceptibility of the Ti64 BM to failure, as compared to the Ti6242 BM, and is in agreement with the observations in previous studies that have related easy slip transfer to similarly oriented α globules [55,56,57] and concomitant lowering of the tensile and fatigue strength [58].

### 3.3. Fatigue Properties

**Figure 8:**
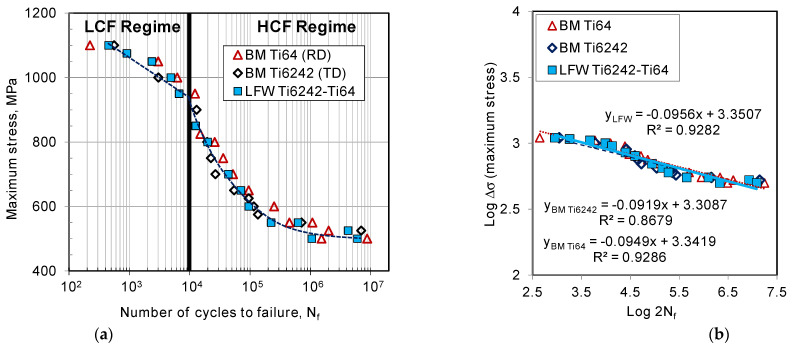
Comparison of fatigue life curves at room temperature and R =  0.1 for the Ti6242 and Ti64 BMs relative to the dissimilar Ti6242–Ti64 linear friction weld in the SRAed condition: (**a**) semi-log scale plot of the maximum stress versus the number of cycles to failure (N_f_) in the (low cycle fatigue) LCF and high cycle fatigue (HCF) regimes and (**b**) double log scale plot of the maximum stress versus the number of reversals to failure (2Nf).

The fatigue life curve (also known as the Wöhler or S-N curve) determines the stress that a material can withstand for a given number of cycles without failure, which is of significance for the design of safety-critical aircraft/aeroengine structures. HCF is characterized by repeated cycling at applied stresses that produce mainly elastic strains in the material, and fracture occurs at a relatively large number of cycles. By contrast, LCF is characterized by repeated cycling at applied stresses that produce plastic deformation in the material, and fracture occurs at a relatively low number of cycles. The fatigue tests were conducted under ambient room temperature conditions and force control with constant amplitude loading at R = 0.1 and a frequency 6 Hz. Both the HCF and LCF behaviors were evaluated for the as-received Ti64 and Ti6242 BMs as well as the SRAed dissimilar Ti6242–Ti64 welds, as shown in the plots of the maximum stress (S) versus number of cycles to failure (N_f_) given in Figure 8a. Comparing the fatigue behavior of the SRAed dissimilar Ti6242–Ti64 welds to the Ti64 and Ti6242 BMs revealed considerable similarities and may be related to failure occurring exclusively in the BMs, which further points to the high quality/integrity and mechanical (tensile and fatigue) resistance of the joints produced by LFW. For instance, in the LCF regime, the dissimilar Ti6242–Ti64 welds in the SRAed state withstood stresses ranging between 950 and 1100 MPa, which was comparable to the fatigue resistance of the Ti64 and Ti6242 BMs. To evaluate the fatigue limit of the dissimilar Ti6242–Ti64 welds in the HCF regime, the S-N_f_ data were plotted on a double logarithmic scale and linear regression analyses were performed on the S-N_f_ data, as given by the trend lines, with their corresponding equations and R^2^ values shown in Figure 8b. Table 4 gives the fatigue limit determined from the linear regression analyses at 10^7^ cycles for the dissimilar alloy welds (450 MPa) as well as the Ti6242 (434 MPa) and Ti64 (445 MPa) BMs. Notwithstanding the reasonably good R^2^ values, the slightly higher fatigue limit determined for the dissimilar Ti6242–Ti64 welds relative to the BMs is likely statistically negligible, but it illustrates the absence of premature/early failures and at least equivalent resistance to Ti6242 and Ti64 under fatigue loading. Thus, to increase statistical rigor for determining the fatigue limit of the dissimilar Ti6242–Ti64 linear friction weld, research methodologies using vibrational HCF testing with statistical analysis [58] may be considered.

**Figure 9:**
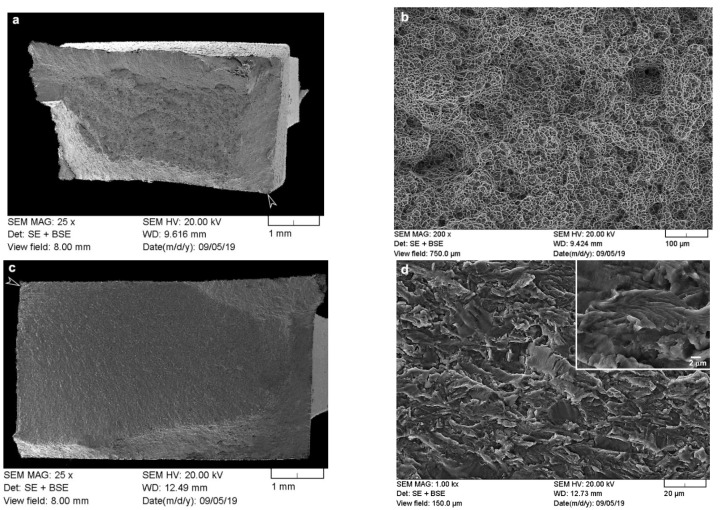
Fatigue fracture surface: (**a**,**b**) LCF overview image with the arrow indicating the failure initiation site and higher -magnification image showing the predominant tensile overload region (Ti64 BM) and (**c**,**d**) HCF overview image with the arrow indicating failure initiation site and higher-magnification image of the crack propagation area (Ti6242 BM) that shows the fatigue striations in the inset image.

As mentioned, failure of the dissimilar Ti6242–Ti64 welds in the SRAed state occurred exclusively in the BMs; however, in the LCF regime, fracture initiated on the Ti64 side, while, in the HCF regime, it was on the Ti6242 side, roughly 3 ± 1 mm away from the weld line for both regimes. The fracture surfaces on both the Ti64 and Ti6242 sides exhibited characteristic features of failure under fatigue conditions. For instance, under LCF conditions, Figure 9a reveals that failure initiated at the surface (on the Ti64 side) and progressed rapidly through the small crack propagation area and failed under tensile overloading conditions, as evidenced by the presence of dimples (microvoids) in the high-magnification image of the fracture surface in Figure 9b. By contrast, under HCF conditions, Figure 9c reveals that the fracture, which also initiated at the surface (on the Ti6242 side), was dominated by the crack propagation region. Figure 9d shows that this region consisted of quasi-cleavage facets with the presence of very fine fatigue striations (as seen in the higher-magnification inset image within Figure 9d).

Fatigue data on titanium alloy linear friction welds remain limited, but some important insights are emerging. It is reasonably accepted that fatigue resistance in titanium alloys is influenced by a hierarchical set of three structural factors: defects, microstructure and residual stresses. Thus, when defects are present, these will have an overriding influence on the fatigue properties that is irrecoverable through microstructural modifications (the second level in the hierarchy). This explains the observations of García and Morgeneyer [33] in their study of the fatigue performance of Ti6242 linear friction welds that exhibited low fatigue strength values, as well as premature/early failures due to remnant porosity at the joint interface. Recently, Garcia et al. [11] also reported on the detrimental impact of defects—such as oxides, pores and contaminants that remain in the joint and originate from inadequate preparation of the faying surfaces prior to LFW—on the fatigue behavior of dissimilar Ti17–Ti64 linear friction welds that failed prematurely at a very low number of cycles or within the first fatigue cycle. Moreover, their work indicated that in the presence of defects, the application of PWHT to relieve residual stresses or modify the microstructure was ineffective in overcoming the compromised fatigue resistance from poor weld integrity, which aligns precisely with the expected outcome from the structural hierarchy. In the absence of defects, the fatigue behavior of titanium alloys is influenced by (second-level) microstructural aspects, such as the characteristics of the α and β phase constituents, including texture, volume fraction, grain orientation, morphology, size, etc. Previously, Stinville et al. [28] studied the fatigue behavior of Ti64 linear friction welds in the AWed condition to understand the influence of different grain structure orientations: RD and TD. Their integral welds failed in the RD workpiece in the LCF regime and TD workpiece in the HCF regime and a transition stress between 700 and 725 MPa was identified. In the present study, a similar stress transitioning from the RD (≥950 MPa) to the TD (≤850 MPa) workpiece was observed to occur during fatigue testing of the dissimilar Ti6242–Ti64 linear friction welds. This finding also follows the hierarchal order in that the SRA treatment applied to the dissimilar Ti6242–Ti64 linear friction welds for recovering HAZ softening—which would also mitigate residual stresses—did not alter the predominant (second-level) influence of orientation. Here, it is important to mention that, while the only difference in the Ti64 workpieces studied by Stinville et al. [28] was the RD/TD orientation, in the present work, the alloy chemistry and bimodal microstructures were also different between the Ti6242 and Ti64 workpieces. This thus points to the important influence of grain orientation on the fatigue behavior and is reasonably associated with the strong elasto-plastic anisotropy of the hexagonal closed packed (HCP) α phase [59,60]. However, a key difference between the present results and those of Stinville et al. [28] is in the fracture location during cycling. Due to the HAZ softening in their welds, failure of their AWed dissimilarly oriented Ti64 joints occurred within 1 to 2 mm from the weld line (i.e., in the HAZ), especially on the RD side during LCF testing [28]. Though Stinville et al. [28] recognized the lower hardness and degraded cyclic plastic properties of the HAZ in their weldment, they were unable to identify the reasons leading to the performance losses. By contrast, in the present work, it was clearly shown that after SRA of the dissimilar Ti6242–Ti64 linear friction welds, hardness recovery in the HAZ occurs due to metastable β transformation to equilibrated levels of α and β phases. This leads to failure being initiated 3 ± 1 mm from the weld line, which is fully in the Ti64 BM or Ti6242 BM, respectively, in the LCF and HCF regimes. Thus, this research study demonstrates that SRA of the dissimilar Ti6242–Ti64 linear friction welds is effective not only for recovering the hardness but also the cyclic plastic properties, as evidenced by the fatigue failure being initiated on the test specimen surface in the BM region of SRAed joints, rather than in the softened HAZ adjacent to WC in the AWed state as in [28]. Finally, from a material design allowable perspective, the uncompromised fatigue properties of the linear friction welds after SRA (as determined in this work) provide a strong assurance of the quality and performance possible for engineering dissimilar titanium alloy joints to advance tailored designs and products for applications under different service requirements.

## 4. Conclusions

The microstructure and mechanical properties of Ti-6Al-4V (Ti64) and Ti-6Al-2Sn-4Zr-2Mo-0.1Si (Ti6242) linear friction welds were evaluated in the as-welded (AWed) and stress relief annealed (SRAed) states and the following conclusions can be drawn from this study:

1. Dissimilar Ti64–Ti6242 joints manufactured by linear friction welding (LFW) exhibited excellent microstructural and mechanical properties in both the AWed and SRAed states. Peak hardness values were recorded in the weld center due the formation of α′ martensite within the refined transformed prior β grain structure that resulted from hot dynamic recrystallization followed by rapid cooling after LFW. A reduction in the peak hardness by 8% occurred after SRA due to the transformation of α′ martensite to a tempered α + β structure. By contrast, the lower hardness in the heat-affected zones (HAZs) on both the Ti64 and Ti6242 sides of the AWed joints recovered after SRA due to the transformation of soft metastable β—which was retained upon rapid cooling after LFW—to equilibrated α-β fractions after heat treatment. There were no noticeable changes in the TMAZ hardness, though microstructural changes (e.g., transformation of retained metastable β) were observed.
2. The tensile mechanical properties of the AWed and SRAed joints surpassed the AMS specifications for the Ti64 and Ti6242 base metal (BM) properties. Tensile failure of the dissimilar Ti6242–Ti64 linear friction welds occurred exclusively in the Ti64 BM, around 5 ± 1 mm away from the weld line for both the AWed and SRAed states, indicating good weld integrity. The tensile fracture surfaces revealed characteristic ductile features with a dimpled appearance.
3. Analysis of the fatigue behavior of the SRAed welds under both the high cycle fatigue (HCF) and low cycle fatigue (LCF) regimes was comparable with the Ti64 and Ti6242 BMs. In the LCF regime, the dissimilar Ti6242–Ti64 linear friction welds withstood maximum stresses ranging between 950 and 1100 MPa and the failure initiated from the surface at a location roughly 3 ± 1 mm away from the weld line in the Ti64 BM. By contrast, in the HCF regime, failure of the welds initiated also from the surface at a location 3 ± 1 mm away from the weld line, but in the Ti6242 BM region. Furthermore, the fatigue limit of the welds at 10^7^ cycles was 450 MPa, which was slightly higher than the endurance of the BMs in the HCF regime.
4. Examination of the LCF fractures in the Ti64 BM showed fractographic surfaces dominated by a tensile overload region (with dimples). On the other hand, the HCF fractures of the Ti6242 BM showed a large crack propagation area on the fracture surfaces and fine fatigue striations within the quasi-cleavage facets.

## Figures and Tables

**Table 1:** Chemical composition of the Ti6242 and Ti64 base metals (BMs) (wt.%) *.

Alloy	Al	Sn	V	Zr	Mo	Si	Fe	H **	O **	N **	C ***	Ti
Ti6242	6.12	2.18	-	4.35	2.19	0.1	0.1	0.009	0.1	0.01	0.01	Bal.
Ti64	6.08	-		4.35	-	-	0.2	0.003	0.09	0.02	0.02	Bal.

* Wavelength dispersive X-ray fluorescence spectrometry used as per American Society for Testing and Materials, ASTM E539 [41]. ** Inert gas fusion used as per ASTM E1447 [42] and ASTM E1409 [43]. *** Combustion analysis used as per ASTM E1941 [44].

**Table 2:** Comparison of the average hardness in the different regions of the dissimilar Ti6242–Ti64 weld in the AWed and SRAed conditions.

Region	Average Hardness—AWed, HV_0.5_	Average Hardness—SRAed, HV_0.5_	Change after SRA	Percentage (%)
Ti6242 BM	345 ± 8	342 ± 9	Insignificant	0.8
HAZ1	333 ± 6	368 ± 4	Increase	10.5
TMAZ1	370 ± 5	370 ± 15	Insignificant	0
WC	398 ± 3	367 ± 2	Decrease	7.3
TMAZ2	360 ± 3	369 ± 12	Insignificant	2.5
HAZ2	315 ± 2	357 ± 3	Increase	13.3
Ti64 BM	326 ± 4	352 ± 5	Increase	8

**Table 3:** Effect of SRA on the average mechanical properties of the dissimilar Ti6242–Ti64 welds.

Property	AWed	SRAed	Change after SRA	Percentage (%)
YS (MPa)	935 ± 13	862 ± 18	Decrease	7.8
UTS (MPa)	1008 ± 4	967 ± 3	Decrease	4.1
EL (%)	11 ± 0.1	12 ± 0.4	Increase	9.1
Failure location	Ti64 BM	Ti64 BM	None	N/A

**Table 4:** Comparison of the fatigue limit of dissimilar Ti6242–Ti64 welds with that of the Ti6242 and Ti64 BMs at 107 cycles.

	Fatigue Limit at 10^7^ Cycles (MPa)	R^2^
Dissimilar Ti6242–Ti64 welds in SRAed state	450	0.928
Ti6242 BM	434	0.868
Ti64 BM	445	0.929

## Data Availability

Data supporting the findings of this study are available within the article.
